# Supplementary material for: Insights into a water-mediated catalytic triad architecture in CE20 carbohydrate esterases
Source: Nat Commun. 2025 Jul 31;16:7034. doi: 10.1038/s41467-025-62387-5 (PMC12313940; doi:10.1038/s41467-025-62387-5)
Supplement: Supplementary file 1 — Supplementary Information [file 41467_2025_62387_MOESM1_ESM.pdf]

**Supplementary information**

# Insights into a water-mediated catalytic triad architecture in CE20 carbohydrate esterases

Michelle Teune[1,†], Plínio S. Vieira[2,†], Thorben Döhler[1], Gottfried J. Palm[3], Theresa Dutschei[1], Daniel Bartosik[4], Leona Berndt[3], Gabriela F. Persinoti[2], Sandra Maaß[5], Dörte Becher[5], Thomas Schweder[4], Mário T. Murakami[2]*, Michael Lammers[3]*, Uwe T. Bornscheuer[1]*

[1]Department of Biotechnology & Enzyme Catalysis, Institute of Biochemistry, University Greifswald, 17489 Greifswald, Germany
[2]Brazilian Biorenewables National Laboratory (LNBR), Brazilian Center for Research in Energy and Materials (CNPEM), Campinas, São Paulo, Brazil
[3] Department of Synthetic and Structural Biochemistry, Institute of Biochemistry, University of Greifswald, 17489 Greifswald, Germany
[4] Department of Pharmaceutical Biotechnology, Institute of Pharmacy, University of Greifswald, 17489 Greifswald, Germany
[5] Department of Microbial Proteomics, Institute of Microbiology, University of Greifswald, 17489 Greifswald, Germany
[†]These authors contributed equally

*Corresponding authors:

Uwe Bornscheuer, uwe.bornscheuer@uni-greifswald.de

Michael Lammers, michael.lammers@uni-greifswald.de

Mário T. Murakami, mario.murakami@lnbr.cnpem.br

## Supplementary Tables

**Supplementary Table 1: Percentage of sequence similarity between full length enzymes and single domains of investigated CE20 enzymes.** The catalytic domain is only representing the SGNH fold, without the N- and C-terminal sandwich domains.

| Full-length sequence (%) | | | | | | | |
|---|---|---|---|---|---|---|---|
| | BnCE20_I | PpCE20_II | Fl8CE20_II | XacXae_III | SsCE20_IV | SpCE20_V | PsCE20_VI |
| BnCE20_I | - | 38.17 | 40.47 | 36.86 | 36.85 | 42.60 | 33.86 |
| PpCE20_II | 38.17 | - | 46.13 | 38.18 | 36.95 | 38.45 | 35.87 |
| Fl8CE20_II | 40.47 | 46.13 | - | 39.35 | 37.21 | 39.47 | 35.41 |
| XacXae_III | 36.86 | 38.18 | 39.35 | - | 37.95 | 36.70 | 41.40 |
| SsCE20_IV | 36.85 | 36.95 | 37.21 | 37.95 | - | 35.23 | 36.71 |
| SpCE20_V | 42.60 | 38.45 | 39.47 | 36.70 | 35.23 | - | 33.23 |
| PwCE20_VI | 33.86 | 35.87 | 35.41 | 41.40 | 36.71 | 33.23 | - |
| **Catalytic domain only (%)** | | | | | | | |
| BnCE20_I | - | 42.79 | 45.29 | 40.72 | 44.22 | 49.67 | 42.34 |
| PpCE20_II | 42.79 | - | 48.78 | 42.57 | 45.54 | 45.86 | 40.04 |
| Fl8CE20_II | 45.29 | 48.78 | - | 42.21 | 45.52 | 43.24 | 43.24 |
| XacXae_III | 40.72 | 42.57 | 45.21 | - | 43.24 | 43.76 | 38.95 |
| SsCE20_IV | 44.22 | 43.82 | 45.54 | 45.52 | - | 45.66 | 37.95 |
| SpCE20_V | 49.67 | 45.86 | 45.66 | 43.76 | 43.82 | - | 36.95 |
| PwCE20_VI | 37.35 | 38.60 | 40.14 | 43.65 | 43.02 | 38.57 | - |
| **Ancillary domain only (%)** | | | | | | | |
| BnCE20_I | - | 34 | 34.36 | 29.86 | 23.53 | 29.05 | 29.11 |
| PpCE20_II | 34.36 | - | 42.16 | 31.88 | 25.55 | 28.46 | 34.29 |
| Fl8CE20_II | 34 | 42.16 | - | 32.73 | 22.22 | 26.52 | 29.49 |
| XacXae_III | 29.86 | 31.88 | 32.73 | - | 24.84 | 24.29 | 36.84 |
| SsCE20_IV | 31.15 | 25.55 | 29.24 | 24.84 | - | 20.27 | 30.50 |
| SpCE20_V | 29.05 | 28.46 | 26.52 | 24.29 | 20.27 | - | 24.81 |
| PwCE20_VI | 23.53 | 34.29 | 22.22 | 35.42 | 31.5 | 24.81 | - |

**Supplementary Table 2: Kinetic parameters of selected enzymes from all clusters of the SSN using pNP-acetate as a substrate.** For calculation of the kinetic parameters, Hill equation was applied using OriginPro®. All measurements were carried out in technical triplicates. Mean values were calculated and standard deviations are shown in grey. Source data are provided as a Source Data file.

| Enzyme | BnCE20_ | PpCE20_II | Fl8CE20_II | SsCE20_IV | SpCE20_V | PsCE20_VI |
|---|---|---|---|---|---|---|
| $K_M$ [mmol $L^{-1}$] | 0.69 | 0.43 | 0.62 | 1.65 | 2.46 | 0.56 |
| | ± 0.10 | ± 0.09 | ± 0.37 | ± 0.28 | ± 0.19 | ± 0.05 |
| $k_{cat}$ [$s^{-1}$] | 52.91 | 114.74 | 9.70 | 13.29 | 749.92 | 11.14 |
| | ± 2.09 | ± 17.01 | ± 2.86 | ± 0.94 | ± 31.41 | ± 0.30 |
| $k_{cat}/K_M$ [mmol $L^{-1}$ $s^{-1}$] | 76.68 | 266.84 | 18.1 | 8.05 | 304.85 | 19.89 |
| | ± 20.9 | ± 189 | ± 4,45 | ± 3.36 | ± 165.32 | ± 6 |

**Supplementary Table 3: Data collection and data refinement statistics for crystallographic data.** Values in parenthesis represent the higher resolution shell.

| | Fl8CE20_II | PpCE20_II |
|---|---|---|
| **PDB Code** | 9H4U | 9EGA |
| **Data collection** | | |
| Space group | $P\ 2_1 2_1 2_1$ | $P\ 1\ 2_1\ 1$ |
| Cell dimensions | | |
| *a*, *b*, *c* (Å) | 96.66, 120.04, 141.55 | 56.13, 125.14, 108.69 |
| α, β, γ (°) | 90, 90, 90 | 90, 104.71, 90 |
| Resolution (Å) | 50–1.54 (1.63 – 1.54) | 50.00 – 1.35 (1.43 – 1.35) |
| $R_{meas}$ | 0.107 (2.532) | 0.08 (1.29) |
| $I/\sigma(I)$ | 12.6 (0.8) | 12.64 (1.27) |
| $CC_{1/2}$ (%) | 0.998 (0.396) | 99.9 (59.9) |
| Completeness (%) | 96.5 (78.4) | 99.2 (95.6) |
| Redundancy | 12.5 (9.0) | 6.69 (6.10) |

| | | |
|---|---|---|
| **Refinement** | | |
| Resolution (Å) | 50–1.54 (1.58 – 1.54) | 49.72 – 1.35 |
| No. reflections | 220154 | 314310 |
| $R_{work}/R_{free}$ | 0.1697/0.2009 (0.355/0.358) | 0.159/0.192 |
| No. atoms | | |
| Protein | 10,170 | 10025 |
| Ligand/Ion | 103/8 | 30/2 |
| Water | 1105 | 1836 |
| *B*-factors (Å) | | |
| Protein | 30.4 | 20.1 |
| Ligand/Ion | 50.0 | 73.3/16.75 |
| Water | 38.4 | 28.12 |
| R.m.s. deviations | | |
| Bond lengths (Å) | | 0.005 |
| Bond angles (°) | | 0.803 |

**Supplementary Table 4: B-factors for Cα-atoms for Ser113, His515 and Asp513, of Fl8CE20_II.**

| | B-factors of Cα-atoms ($Å^2$) | |
|---|---|---|
| amino acid side chain | **chain A** | **chain B** |
| Ser112 | 17.7 | 19.6 |
| His515 | 20.1 | 26.9 |
| Asp513 | 23.2 | 29.7 |
| average | 29.3 | 27.9 |

**Supplementary Table 5: Kinetic parameters of selected mutants of Fl8CE20_II.** For calculation of the kinetic parameters, the Michaelis-Menten equation was applied using OriginPro®. All measurements were carried out in technical triplicates. Mean values were calculated and standard deviations are shown in grey. The enzymatic activity of mutation D513A, H515A and S112A could not be determined. Source data are provided as a Source Data file.

| Variant | $K_M$ [mmol L$^{-1}$] | $k_{cat}$ [s$^{-1}$] | $k_{cat}/K_M$ [mmol L$^{-1}$ s$^{-1}$] |
|---|---|---|---|
| **wt** | $6.23 \cdot 10^{-1}$<br>$\pm 3.65 \cdot 10^{-1}$ | 9.70<br>± 2.86 | 18.09<br>± 4.45 |
| **D513N** | $2.92 \cdot 10^{-1}$<br>$\pm 1.81 \cdot 10^{-1}$ | 1.91<br>± 0.89 | 7.20<br>± 1.08 |
| **D513E** | $2,30 \cdot 10^{0}$<br>$\pm 2.36 \cdot 10^{0}$ | 0.18<br>± 0.14 | 0.43<br>± 0.50 |
| **D513Q** | $9.89 \cdot 10^{0}$<br>$\pm 4.48 \cdot 10^{0}$ | 1.64<br>± 0.62 | 0.18<br>± 0.03 |
| **K370M** | $2.92 \cdot 10^{-1}$<br>$\pm 2.52 \cdot 10^{-2}$ | 3.78<br>± 0.96 | 12.9<br>± 2.82 |
| **R313E** | $2.97 \cdot 10^{-1}$<br>$\pm 7.39 \cdot 10^{-2}$ | 4.67<br>± 1.18 | 15.7<br>± 0.38 |
| **R313Q** | $4.24 \cdot 10^{-2}$<br>$\pm 2.07 \cdot 10^{-2}$ | 1.25<br>± 0.36 | 33.0<br>± 7.65 |
| **S434A** | $2.14 \cdot 10^{-1}$<br>$\pm 1.86 \cdot 10^{-2}$ | 4.48<br>± 0.54 | 20.9<br>± 1.49 |
| **Y343N** | 2.20<br>$\pm 0.502 \cdot 10^{-1}$ | 2.72<br>± 3.46 | 12.7<br>± 1.41 |

# Supplementary Figures

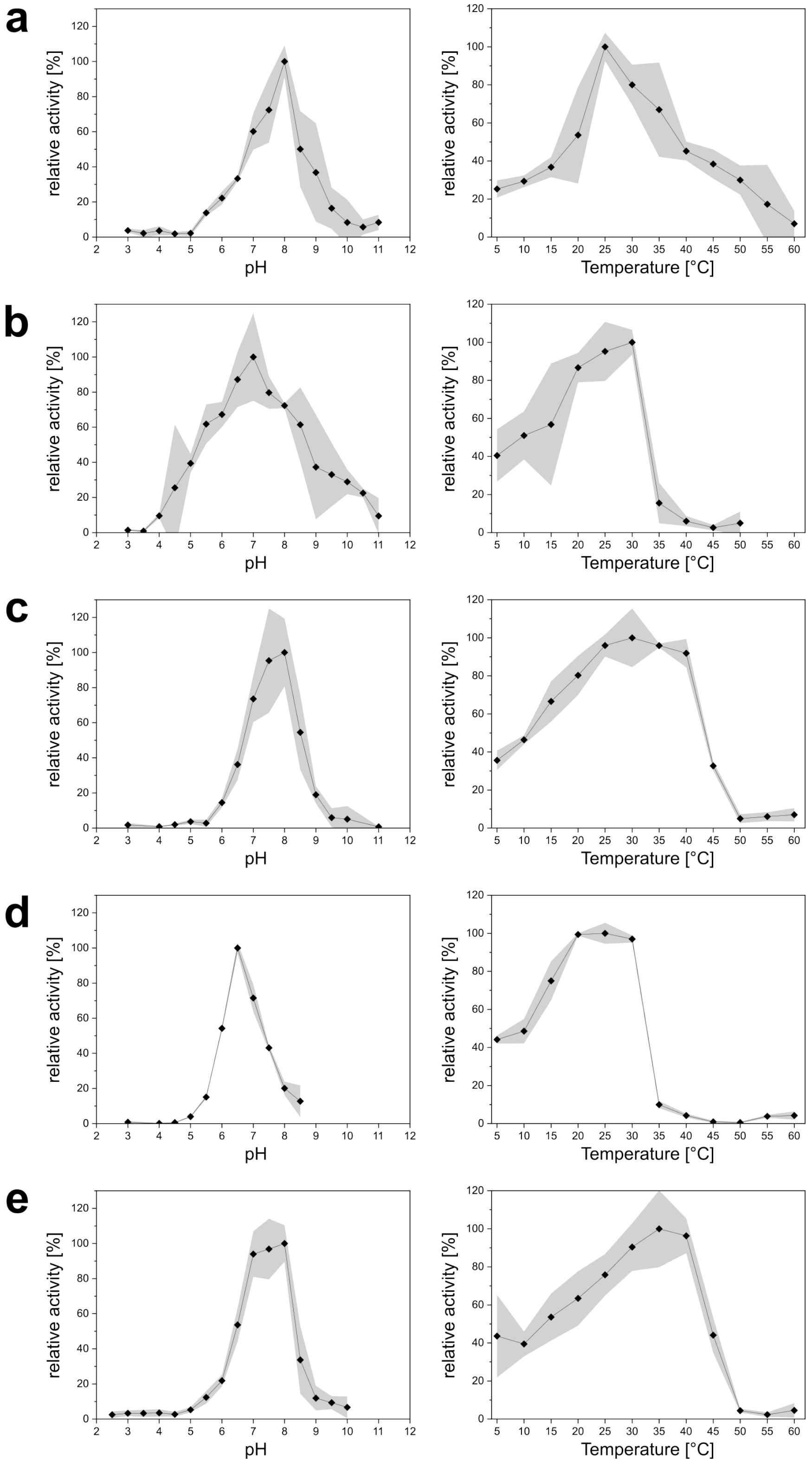


**Supplementary Figure 1: pH and reaction temperature profiles of PpCE20_II (a), BnCE20_I (b), PwCE20_VI (c), SsCE20_IV (d) and SpCE20_V (e) from the SSN.** Activity investigations were carried out at RT using 1 mM *p*NP-acetate. The calculation of relative activity was normalized to the highest measured activity. All measurements were carried out in technical triplicates. Mean values were calculated and standard deviations are shown in grey. Source data are provided as a Source Data file.

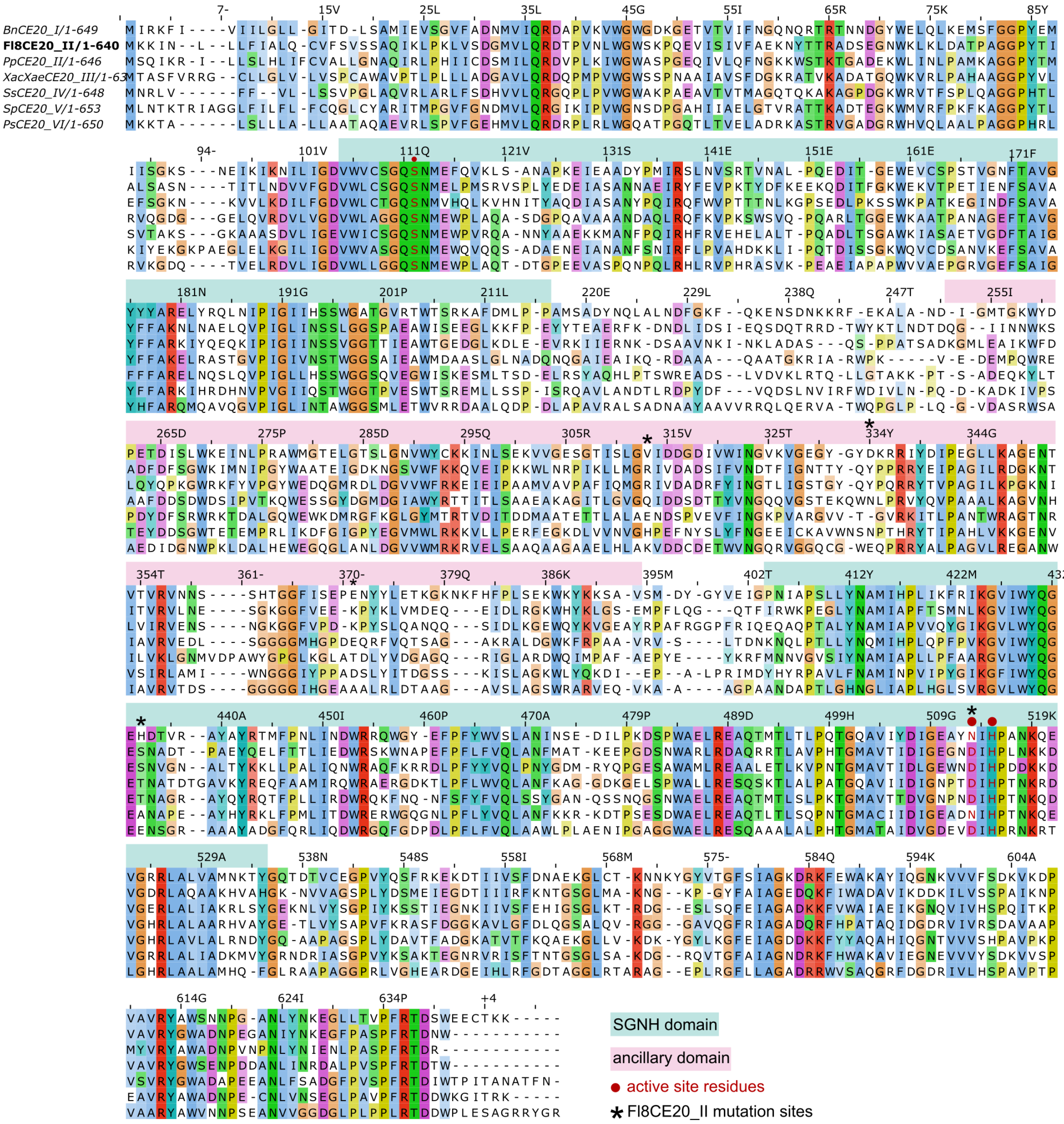


**Supplementary Figure 2: Alignment of selected sequences from the SSN.** Alignment was performed using the seven shown representatives of the SSN with Jalview[1], Tcoffee[2] default settings. Amino acids are coloured according to their properties, hydrophobic ones as blue (ACFHILMVWY), negatively charged as violet (DE), positively charged as red (KR), polar as green (STQN), glycine in orange (G) and proline in yellow (P). Intensities varies according to their conservation. SGNH (light green) and Ancillary domain (light pink) are coloured according to the Fl8CE20_sequence. Active site (red font) and targeted mutation sites (asterisk) are highlighted.

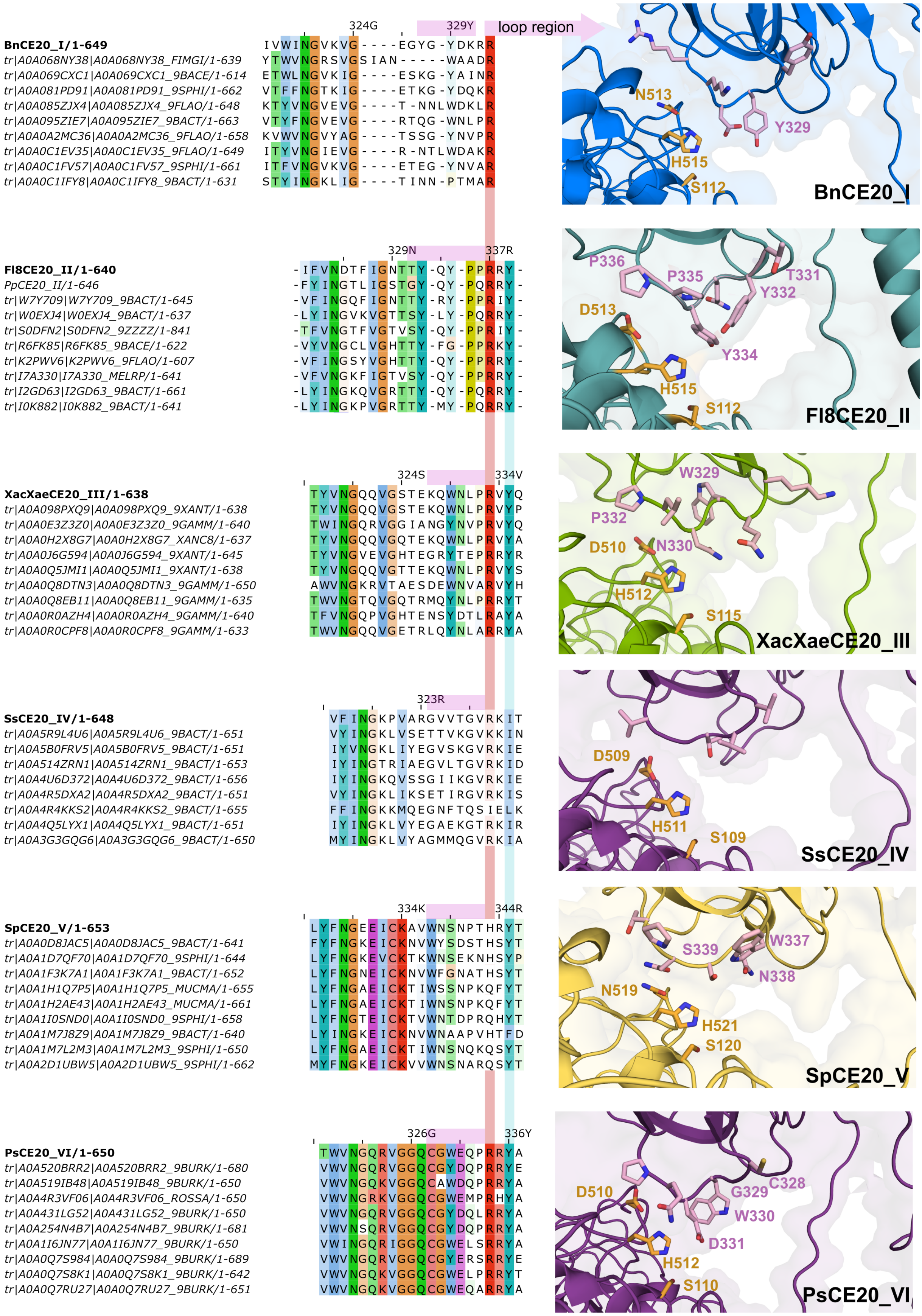


**Supplementary Figure 3: Cluster based sequence alignment of the loop region and structural representation of the conserved residues near the active site.** Alignment was performed using all sequences from each cluster with Jalview[1], Tcoffee[2] default settings. Amino

acids are coloured according to their properties, hydrophobic ones as blue (ACFHILMVWY), negatively charged as violet (DE), positively charged as red (KR), polar as green (STQN), glycine in orange (G) and proline in yellow (P). Intensities varies according to their conservation. The representatives of each cluster were set as a reference. For cluster II, Fl8CE20_II was set as a reference. For reasons of visualization only ten sequences are shown for each cluster. For comparability the alignment of the clusters was performed using a conserved Arg (red vertical line) and Tyr (blue vertical line). For the structural representation, AlphaFold3 models of each representative (crystal structure for Fl8CE20_II) were used. Active site residues are represented in orange. The side chains of the loop region are shown as pink sticks. Conserved residues within the loop region are labelled.

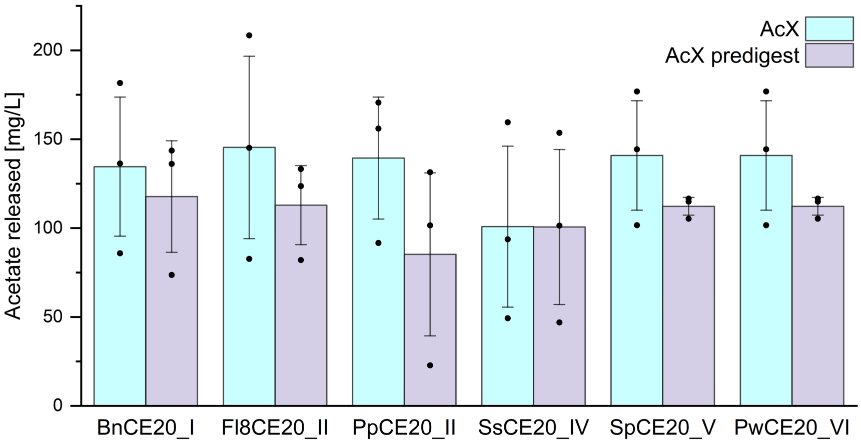


**Supplementary Figure 4: Deacetylase activity of SSN cluster representatives towards acetylated xylan.** Activity was determined towards acetylated xylan polysaccharide (AcX) and oligosaccharides (AcX predigest). All measurements were carried out in technical triplicates. Mean values were calculated and standard deviations are shown as bars. Source data are provided as a Source Data file.

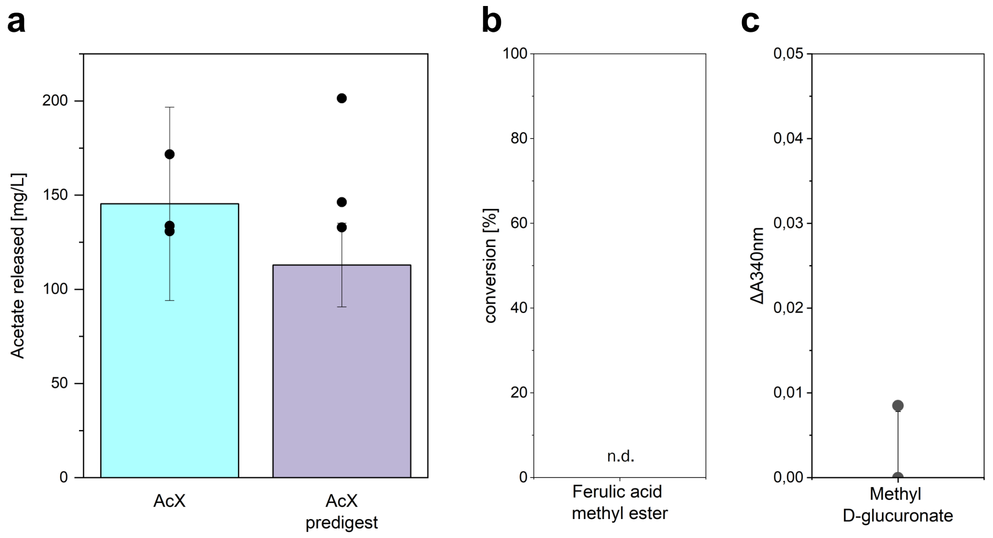


**Supplementary Figure 5: Investigation of xylanolytic activity of Fl8CE20_II.** Activity was determined towards (**a**) acetylated xylan polysaccharide (AcX) and oligosaccharides (AcX predigest) (**b**) ferulic acid methyl ester and (**c**) glucuronic acid methyl ester. This confirms acetyl xylan esterase activity previously described.[3] Values presented in (**a**) are also presented in Supplementary Figure 4 for Fl8CE20_II. No product formation could be detected for ferulic acid methyl ester biotransformation (n.d.: not detectable) and glucuronic acid methyl ester. All measurements were carried out in technical triplicates. Mean values were calculated and standard deviations are shown as bars. Source data are provided as a Source Data file.

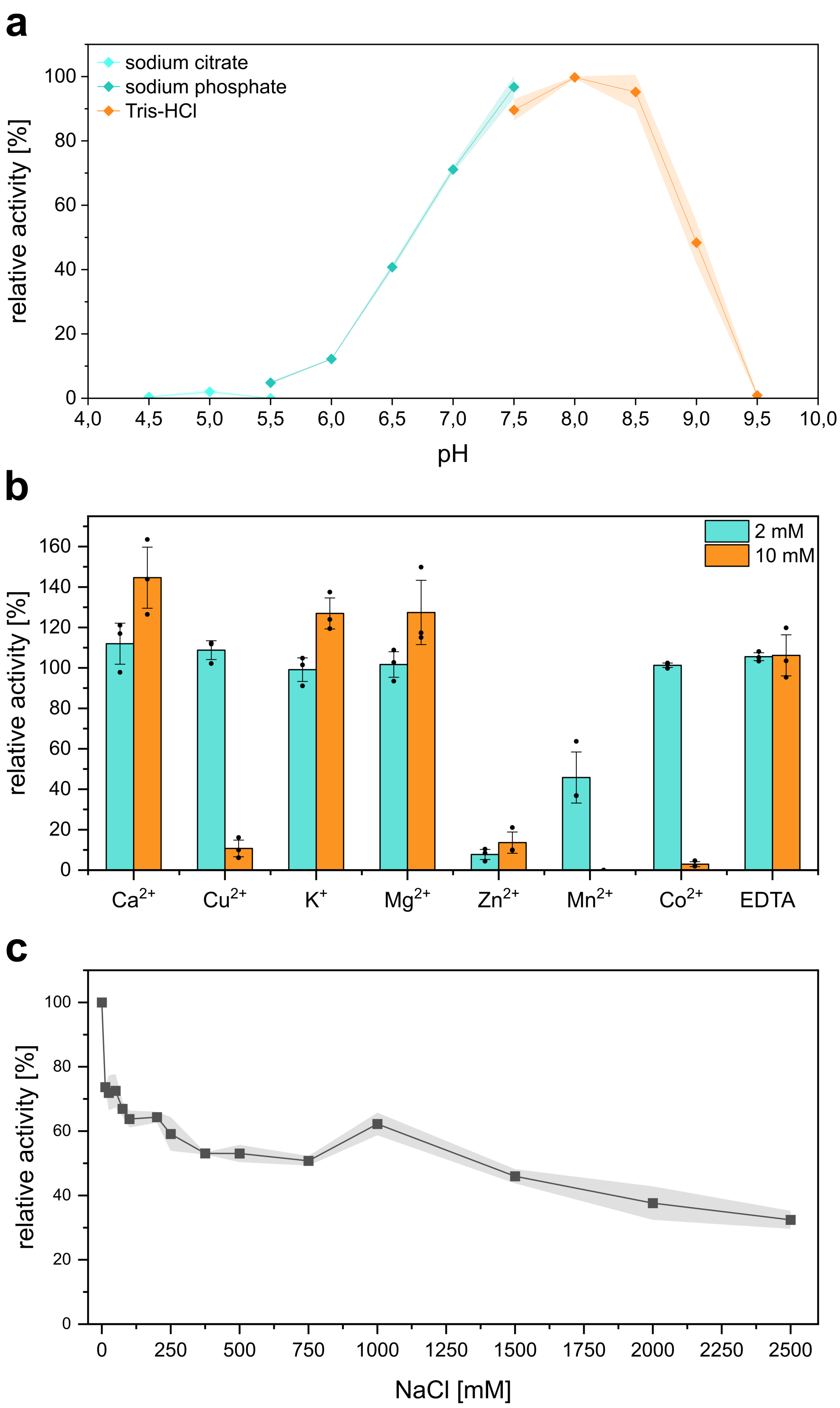


**Supplementary Figure 6: pH profile (a), tolerance towards different metal ions (b) and salinity (c) of Fl8CE20_II.** Activity investigations were carried out at RT using 1 mM *p*NPA. The calculation of relative activity is normalized by the control without additional ions (B) or by the highest measured activity (C). All measurements were carried out in technical triplicates. Mean values were calculated and standard deviations as bars or areas. Source data are provided as a Source Data file.

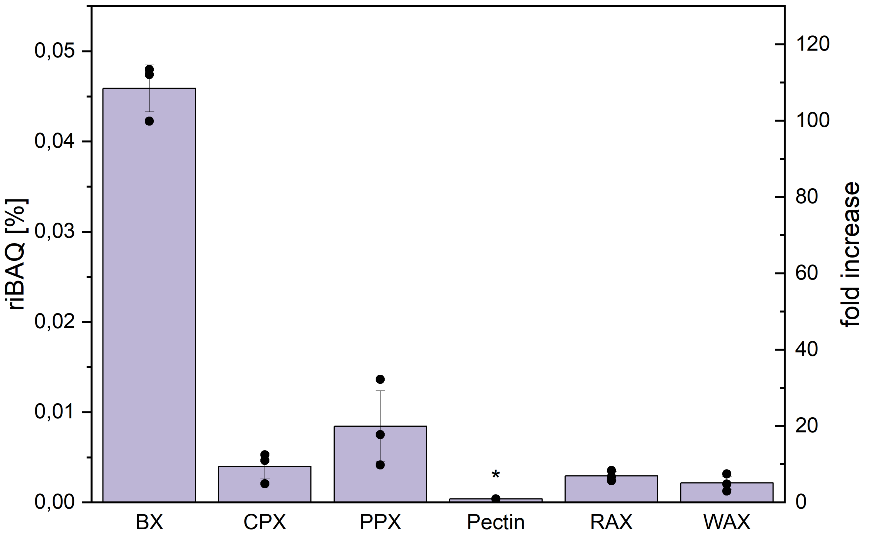


**Supplementary Figure 7: Abundancies (riBAQ) of Fl8CE20_II in proteogenomic datasets upon growth of *Flavimarina* sp. Hel_I_48 different xylans and pectin from apple.** Data derives from a previous study by Dutschei et al.[3] and was visualized to show the fold increase of Fl8 abundancy in the proteome of *Flavimarina* sp. Hel_I_48 growing on different xylans compared to pectin as an acetylated polysaccharide negative control. *As Fl8 seems to be rarely expressed upon growth on pectin, only in one replicate the protein could be found, therefore no replicates could be performed. All measurements were performed in technical triplicates, mean values and standard deviations are presented. Source data are provided as a Source Data file.

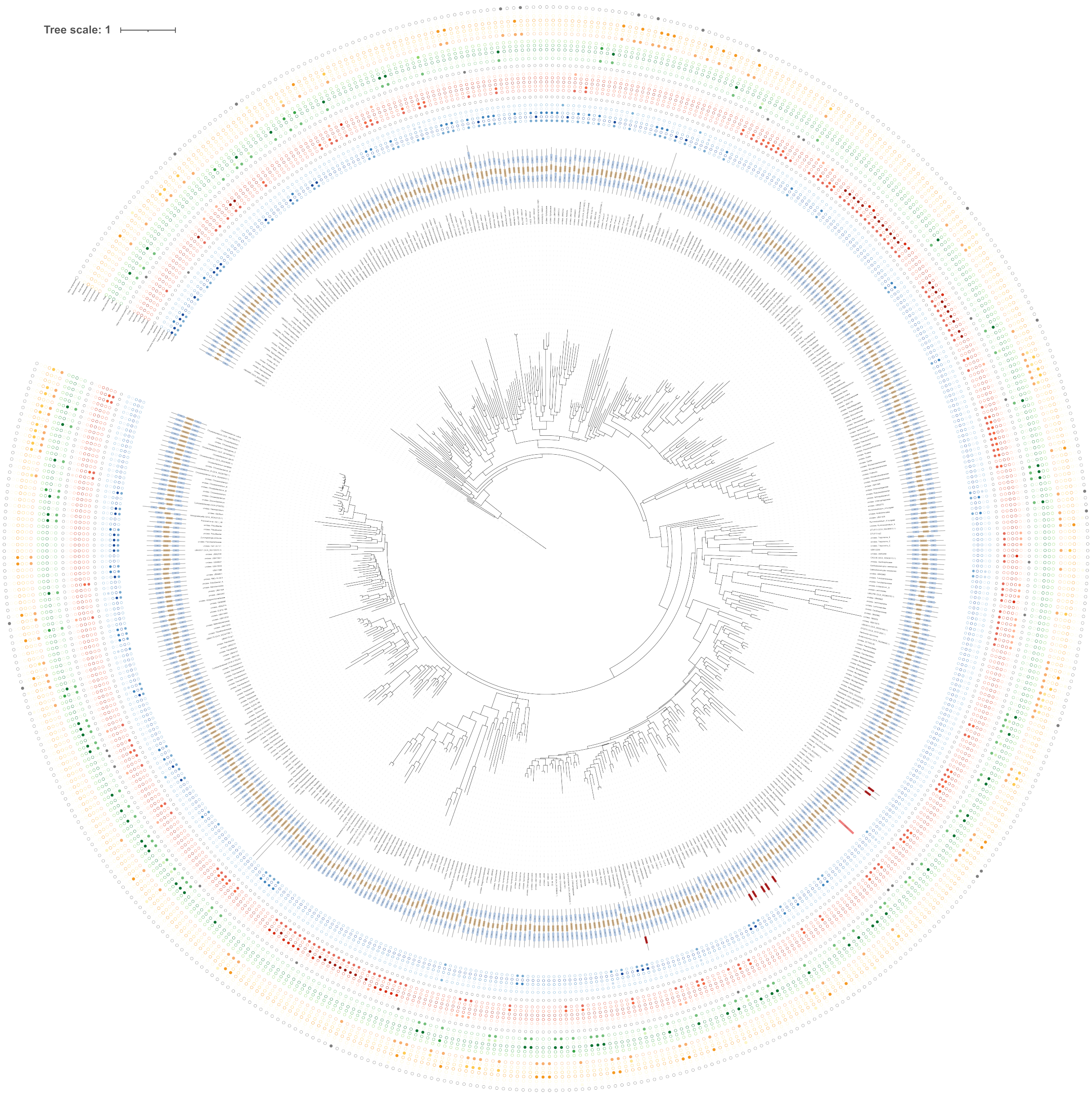


**Supplementary Figure 8: Phylogenetic tree analyses of FI8CE20_II homologues.** The homologues were identified based on the FI8CE20_II domain architecture consisting of the catalytic domains (PF03629.21, SGNH/SASA) and the binding domain PF02837.21, Glyco_hydro_2_N). 502 sequences from 455 genomes were identified.

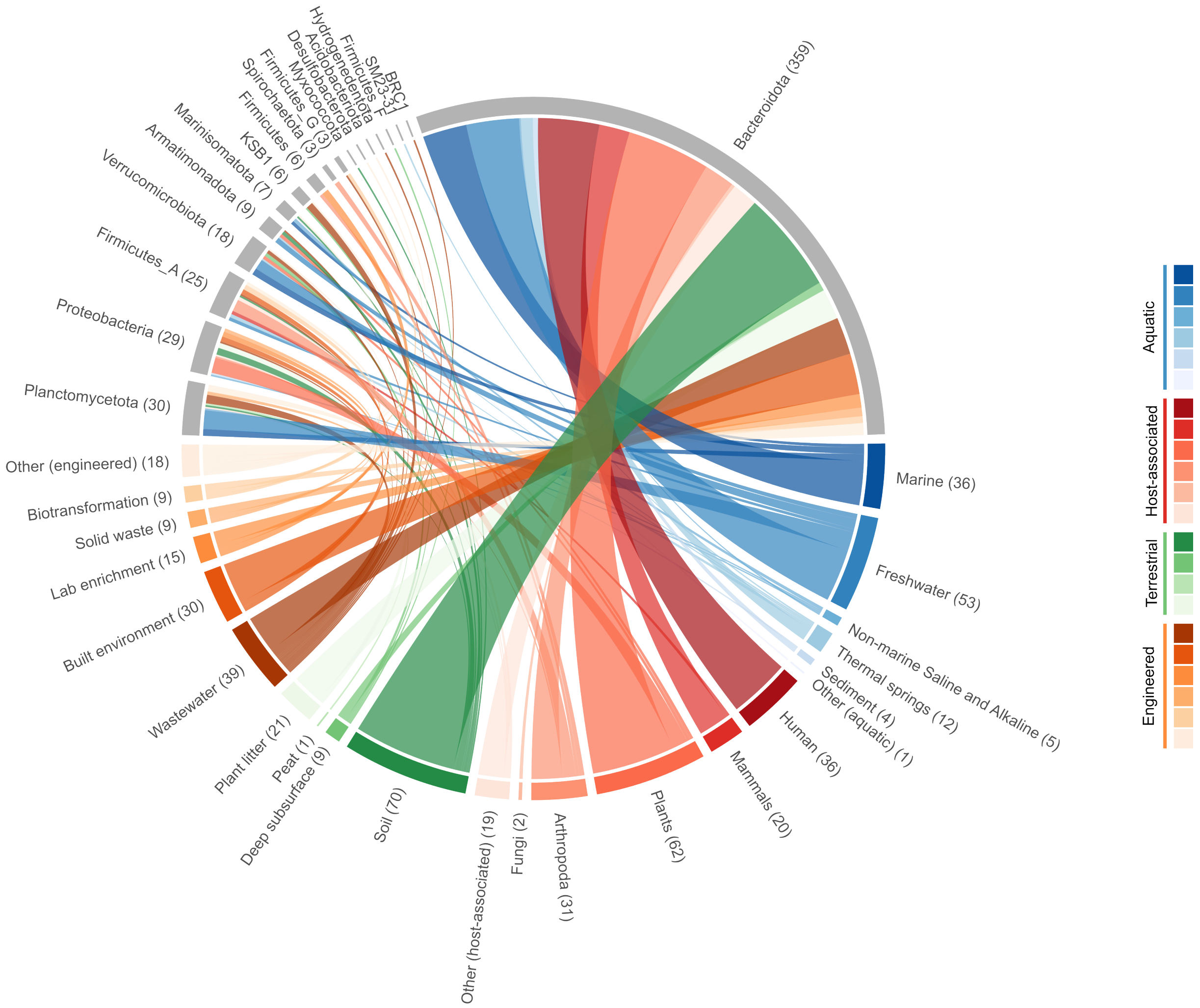


**Supplementary Figure 9: Distribution of the originating habitats of the 502 FI8 homologues grouped in aquatic, host associated, terrestrial and engineered.** The most prominent sub biomes are soil, freshwater, plants, wastewater, human and marine.

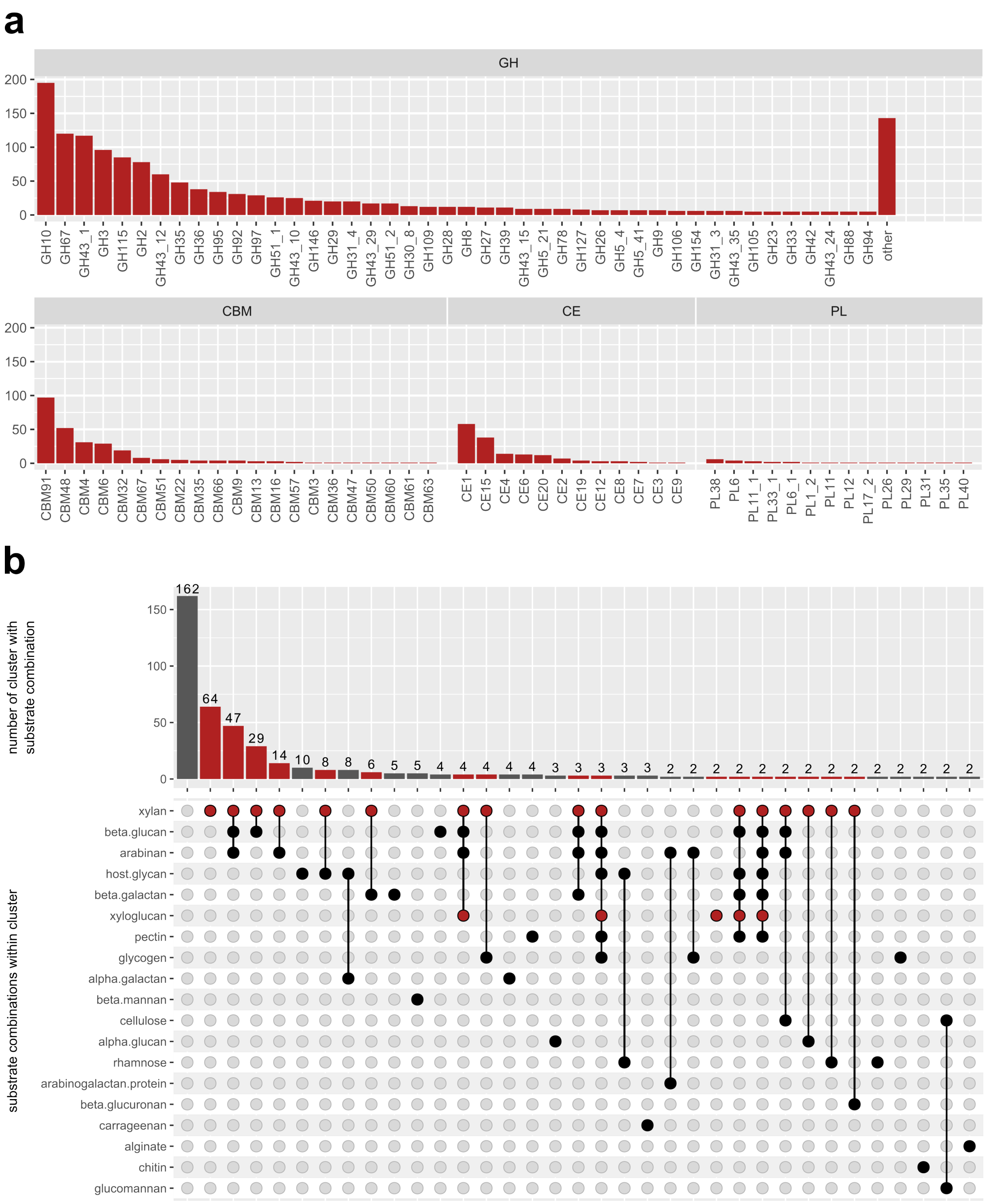


**Supplementary Figure 10: Genomic neighbourhood analyses of co-occurring CAZymes of ten genes up-and downstream of the Fl8 homologues (a) and the predicted substrates of the gene clusters using dbCAN-sub (b).** Substrates that had only a single sequence assigned were excluded. 259 sequences could be assigned to a substrate while 162 sequences could not be predicted. 106 of them showed no co-occurring CAZymes. 194 of the 259 assigned sequences (ca. 75%) were predicted to address xylans, only 13 (5%) xyloglucans. Source data are provided as a Source Data file.

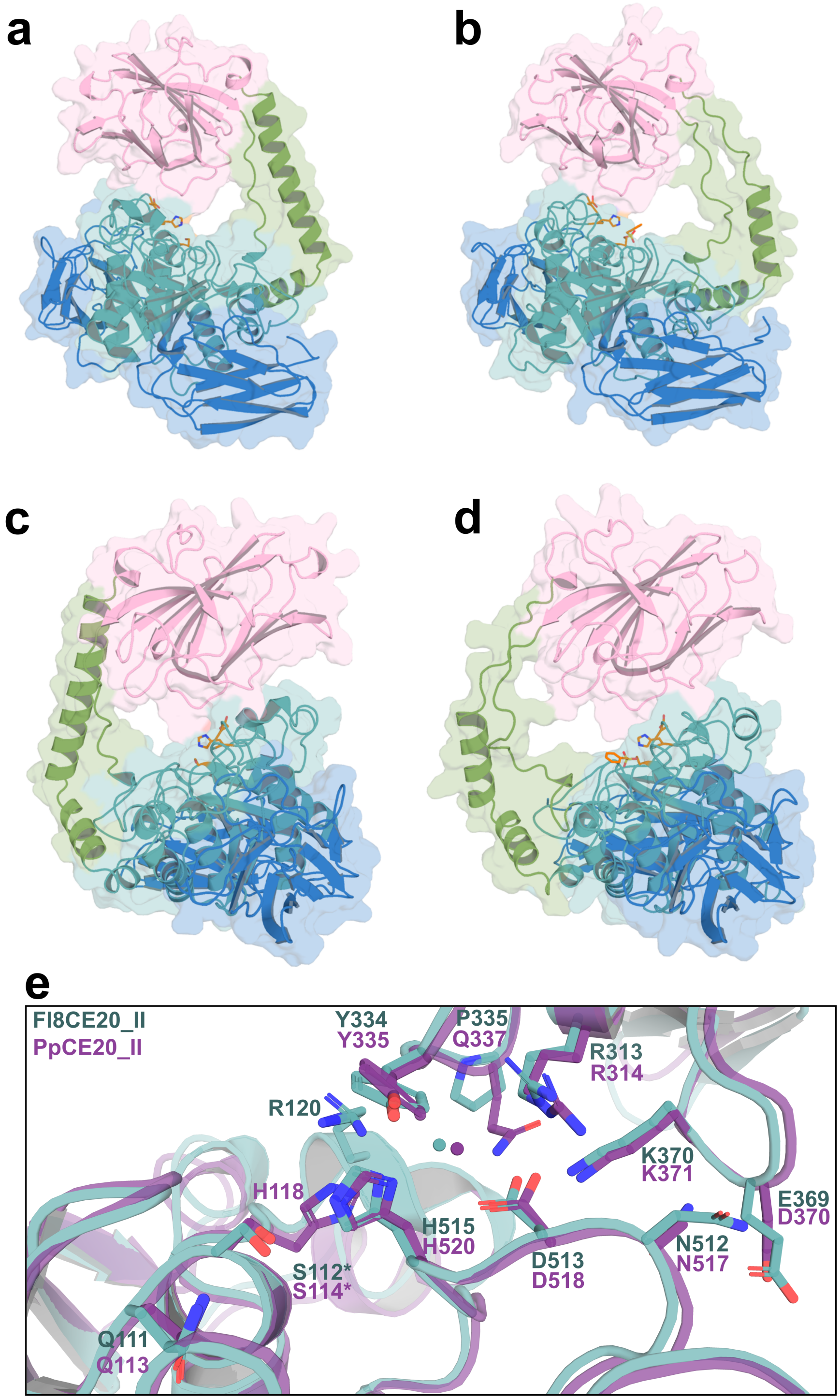


**Supplementary Figure 11: Crystal structures and active sites of Fl8CE20_II and PpCE20_II.** Cartoon and surface representation of the catalytic domain (shades of blue) and the ancillary domain (light pink). Both domains are connected by a linker motif (green), that forms a hoop. Catalytic dyad and anchor residues are represented as orange sticks. Front view and side view of Fl8CE20_II (a and c) and PpCE20_II (b and d). The alignment of the active site of Fl8CE20_II (blue) and PpCE20_II (violet) shows high similarity in their overall structure and conserved residues (e). The crystal structures of Fl8CE20_II (9H4U) and PpCE20_II (9EGA) are available at PDB. *Upon purification the catalytic serine of PpCE20_II was PMSD modified. Upon crystallization the catalytic serine of Fl8CE20_II was cacodylated. For better visualization of the active site, the modifications were replaced by serine.

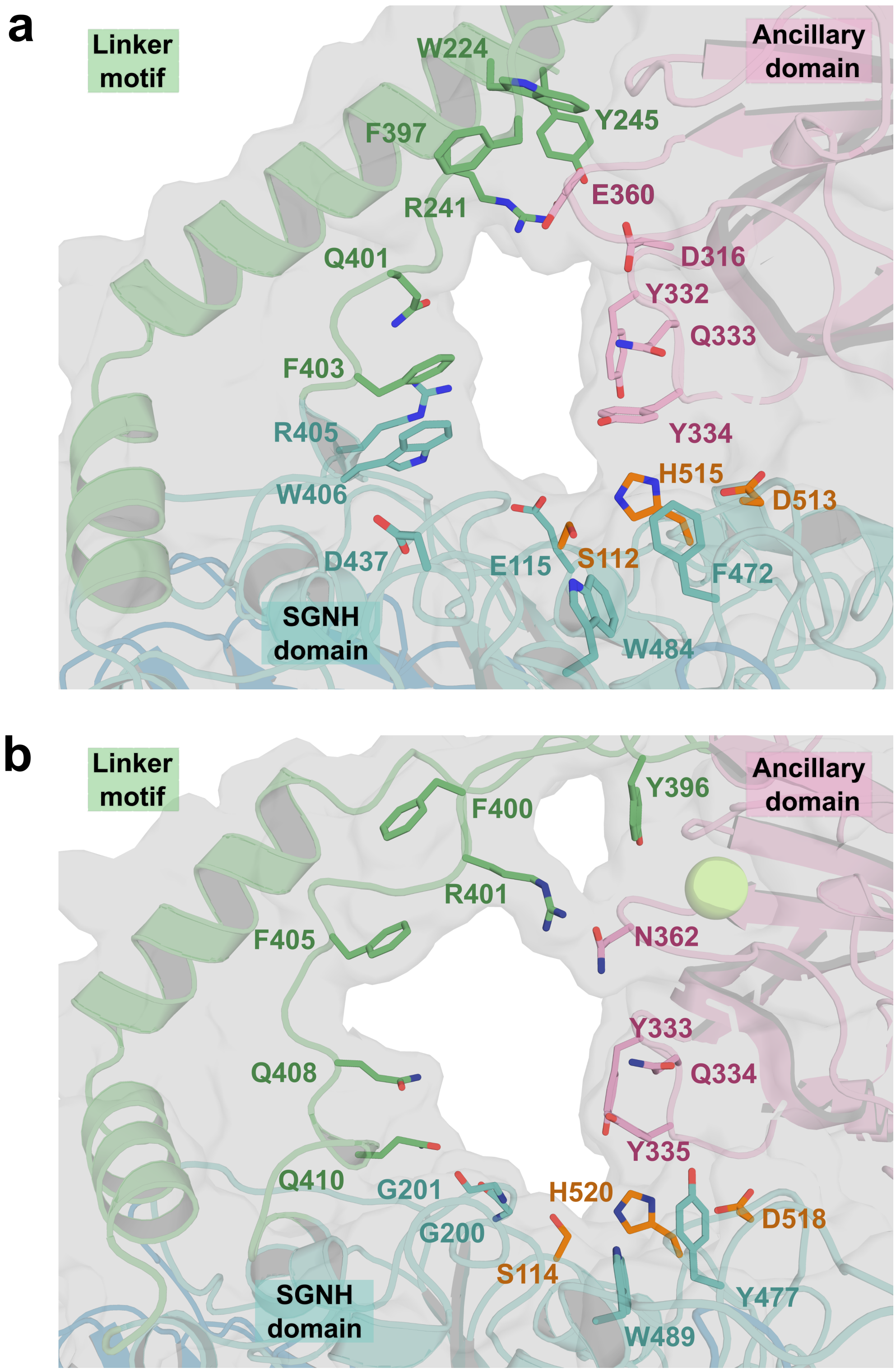


**Supplementary Figure 12: Hoop formation in the crystal structure of Fl8CE20_II (a) and PpCE20_II (b).** Cartoon and surface representation of the catalytic SGNH domain (shades of blue) and the ancillary domain (light pink). Both domains are connected by a linker motif (green), that forms a hoop. Diluted catalytic triad residues are represented as orange sticks.

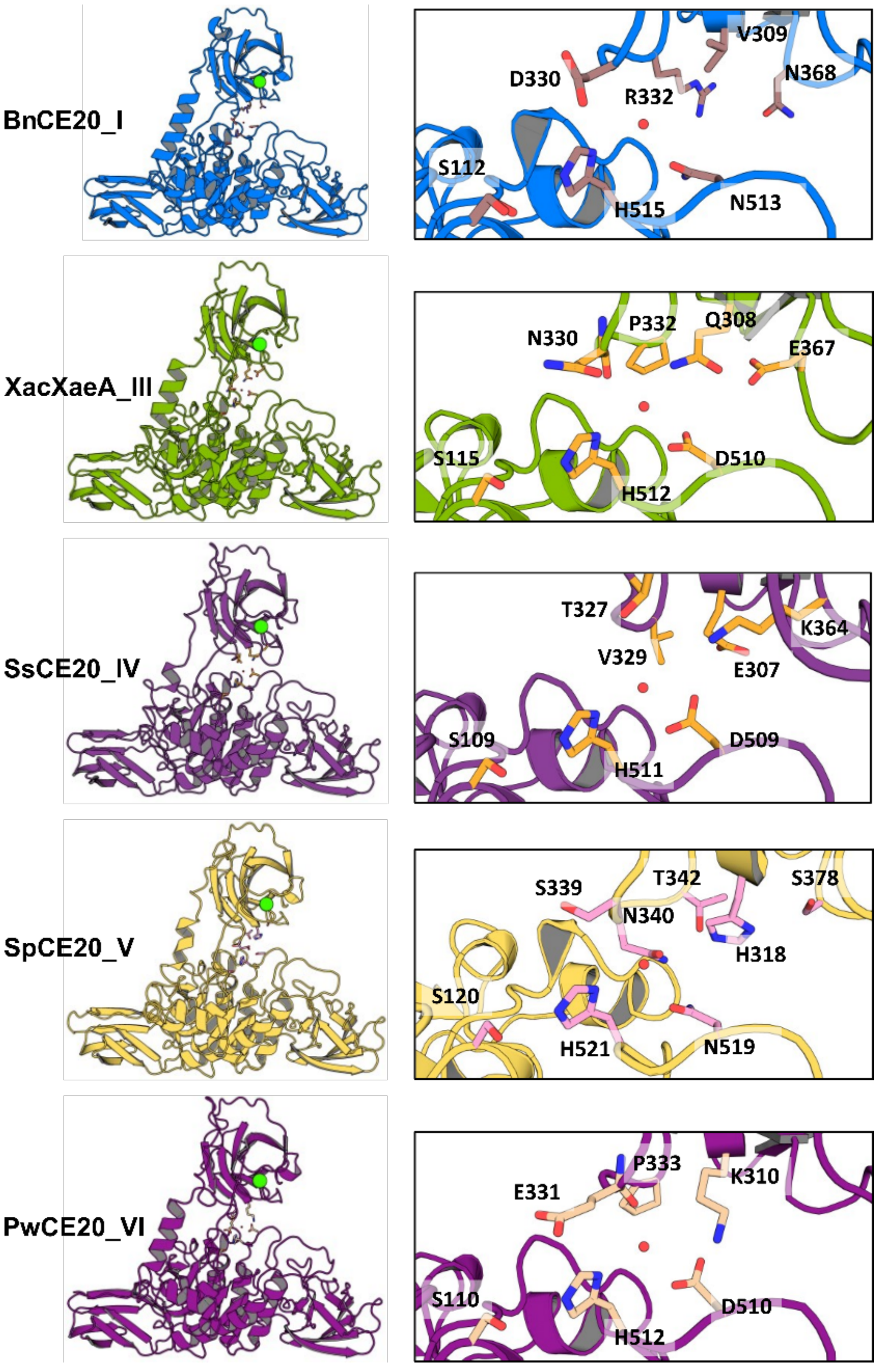


**Supplementary Figure 13: Alphafold2 models and active sites of selected CE20 enzymes from different clusters of the SSN.[4]** Cartoon representation of the Alphafold2 models. The active site and selected residues of the ancillary domain are represented as sticks. The coordinated water molecule (red dot) and the $Mg^{2+}$ ion (green sphere) of the diluted catalytic triad was positioned through superimposition with the crystal structure of PpCE20_II.

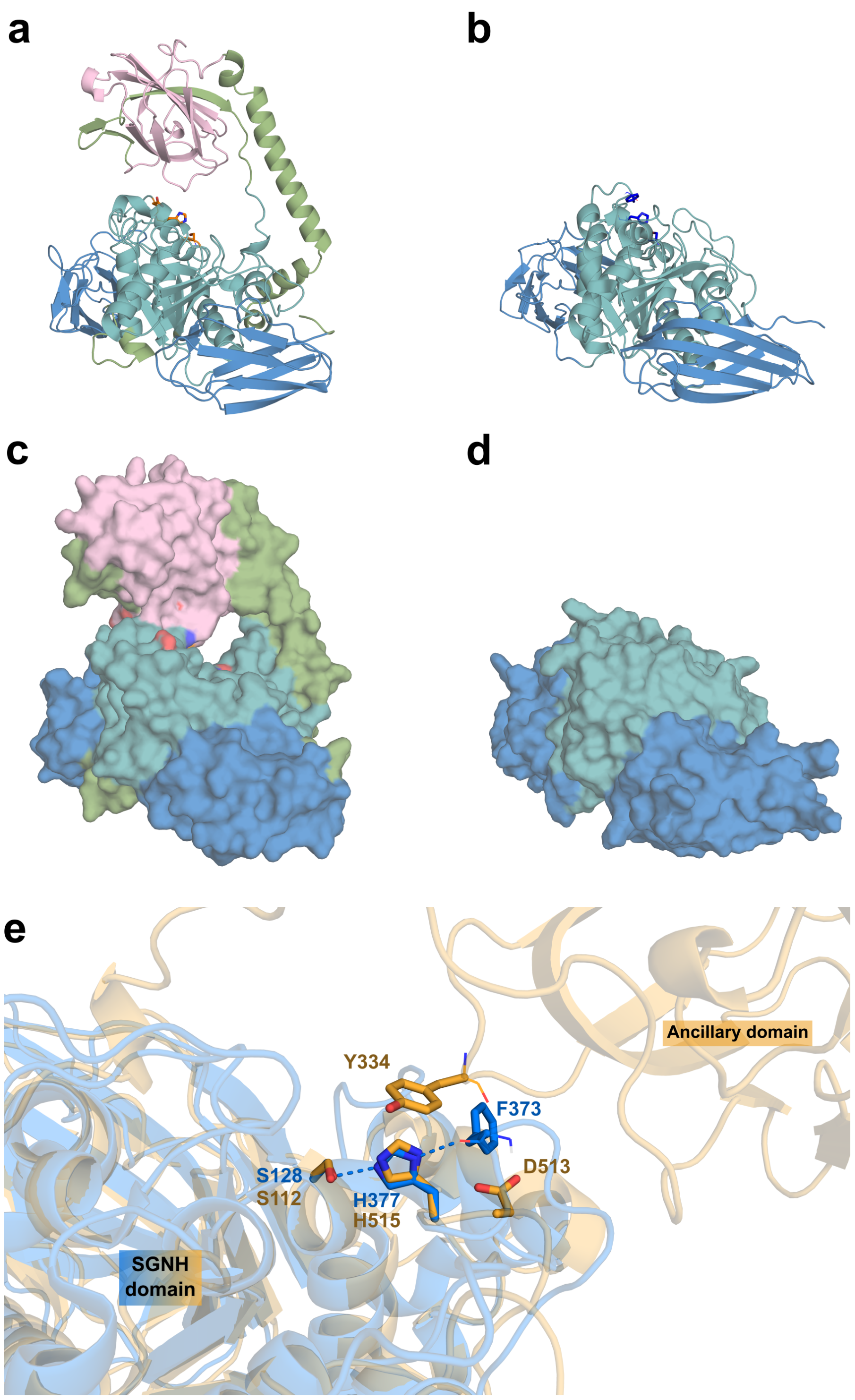


**Supplementary Figure 14: Comparison of CE20 enzyme Fl8CE20_II with ancillary domain and the eukaryotic enzyme 8F9O[5] without ancillary domain.** Cartoon (Fl8CE20_II: **A**, 8F9O: **B**) and surface (Fl8CE20_II: **C**, 8F9O: **D**) representation of the catalytic domain (shades of blue) and the ancillary domain (light pink). Both domains are connected by a linker motif (green), that forms a hoop. Relevant active site residues of Fl8CE20_II are represented as orange. The catalytic Ser-His dyad of 8F9O is shown as blue sticks. (**E**) Overlap of the active site of Fl8CE20 (orange) and 8F9O (blue). Dashed lines represent measurements between catalytic residues of 8F9O**.**

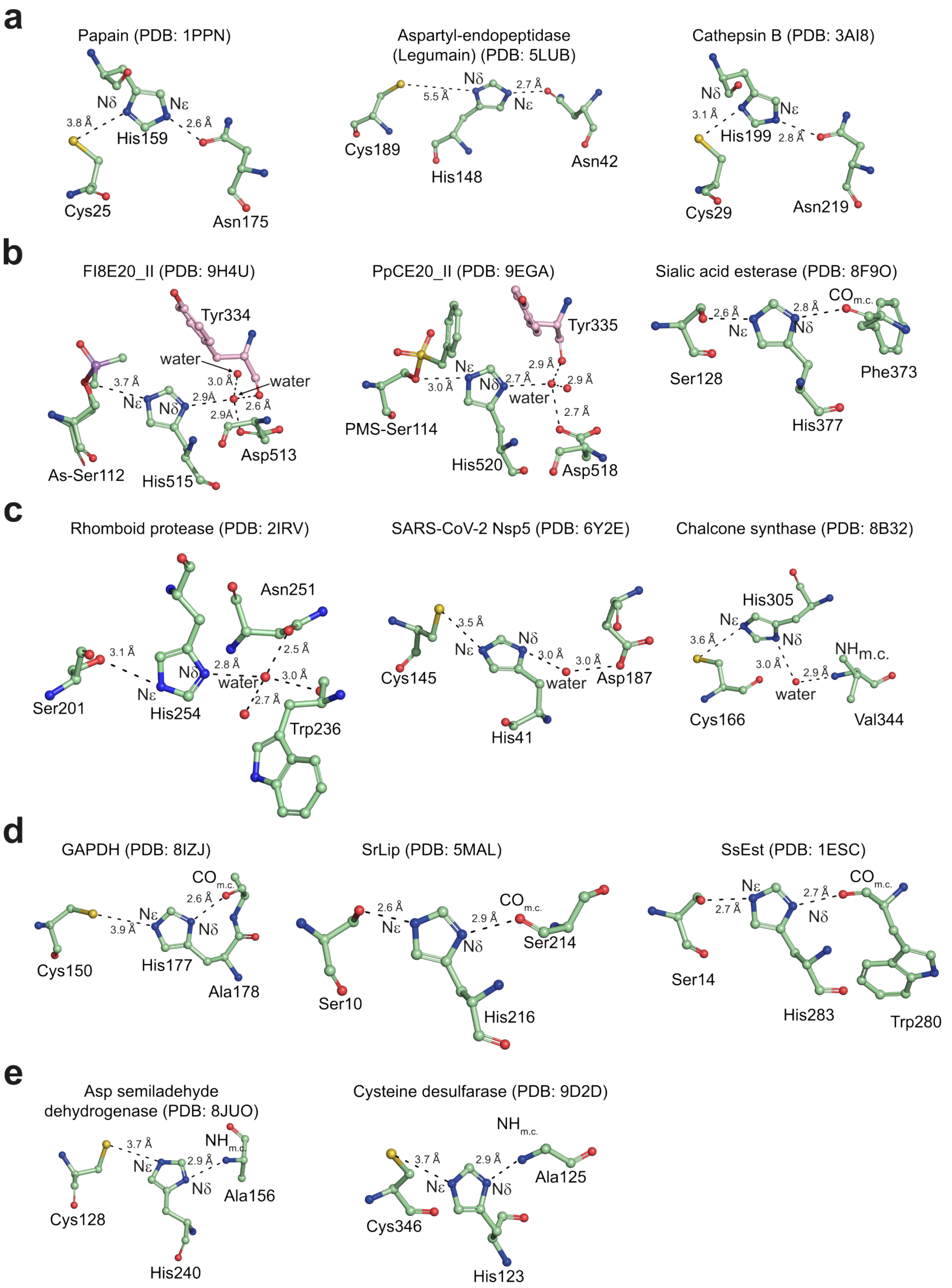


**Supplementary Figure 15: Active site architecture of representative enzymes discussed in this study with various molecular entities at the third position of the catalytic triad.** (**a**) Enzymes using Cys-His-Asn for catalysis. Shown is papain from *Carica papaia* (PDB: 1PPN), Aspartyl-endopeptidase (Legumain) from *Leishmania torentulae* (PDB: 5LUB) and cathepsin B from *Homo sapiens*. (**b**) CE20 enzymes with SGNH hydrolase domain. Shown are the active sites from FI8CE20_II from *Flavimarina* sp. Hel_I_48 (PDB: 9H4U) and PpCE20_II from *Pedobacter psychrotolerans* (PDB: 9EGA) of the structures solved here using a water to position the His base and of dog sialic acid esterase (PDB: 8F9O) using a main

chain carbonyl of Phe373 to position the His base. (**c**) Other enzymes potentially using a water molecule in the active site architecture. Shown are the active sites for rhomboid protease from *Escherichia coli* K12 (PDB: 2IRV), SARS-CoV-2 Nsp5 (3C-like proteinase; PDB: 6Y2E) and chalcone synthase from *Hordeum vulgare* (PDB: 8B32) In chalcone synthase the water is coordinated by two main chain NH groups from Ser343 and Val344. Only Val344 is shown. (**d**) Enzymes using a main chain carbonyl group to position the histidine base. Shown are the active sites for glyceraldehylde-3-phosphate dehydrogenase (GAPDH) from *E. coli* (PDB: 8IZJ), *Streptomyces rimosus* extracellular lipase (SrLip) (PDB: 5MAL) and *Streptomyces scabies* esterase (PBD: 1ESC). (**e**) Enzymes applying a main chain NH group to orient and protonate the His base. Shown is the active site for Asp semialdehyde dehydrogenase from *Porphyromonas gingivalis* (PDB: 8JUO) and from cysteine desulfurase from *E. coli* (PDB: 9D2D). The distances between the side chain or main chain atoms and water molecules are given in Å. For  Fl8CE20_II/PpCE20_II the Tyr334/Tyr335 located in the ancillary domain is coloured in pink.

**a**

**b**

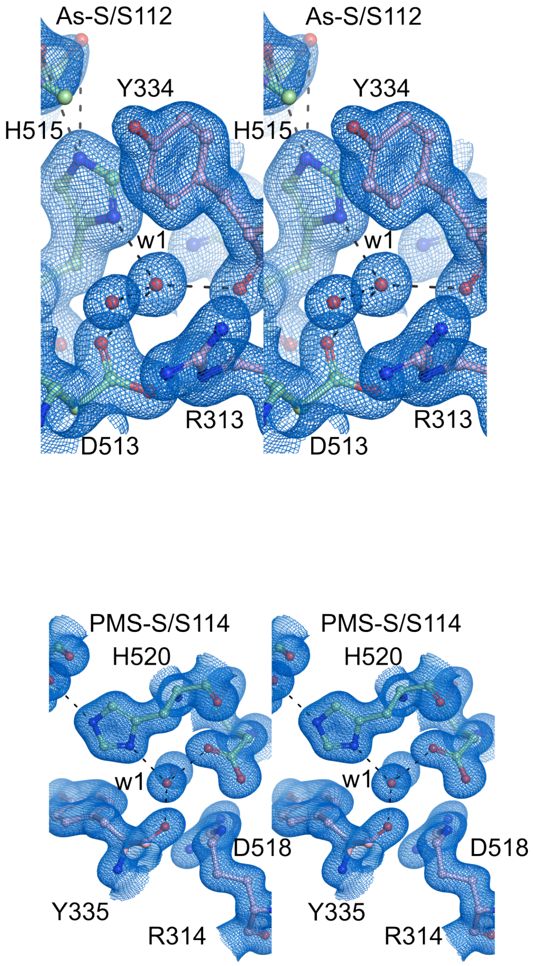


**Supplementary Figure 16: Stereo diagrams showing representative electron density maps of the structures of Fl8CE20_II from *Flavimarina* sp. Hel_I_48 and PpCE20_II from *Pedobacter psychrotolerans*.** The $2F_O$-$F_C$ electron density maps were contoured at $1\sigma$. The closeup shows the active site architecture of catalytic water-mediated catalytic triad. The w1 is the catalytic water is molecule tetrahedrally coordinated by the main chain carbonyl of the Tyr, carboxylate of the catalytic Asp, N$\varepsilon$ of the catalytic base His and by another water molecule.

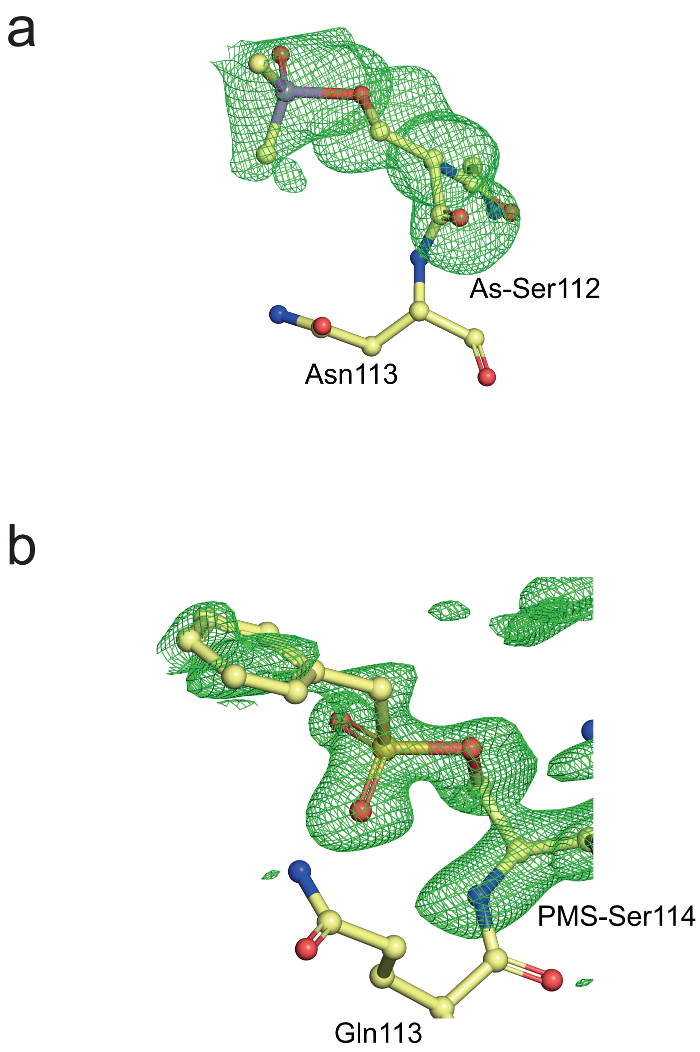


**Supplementary Figure 17: Omit maps of the modified, cacodylated catalytic Ser112 (As_Ser112) of Fl8CE20_II from *Flavimarina* sp. Hel_I_48 (a) and PMS-modified Ser114 (PMS-Ser114) of PpCE20_II from *Pedobacter psychrotolerans* (b).** The $F_O$-$F_C$ omit maps were contoured at $3\sigma$.

## Additional references


1. Waterhouse, A. M., Procter, J. B., Martin, D. M. A., Clamp, M. & Barton, G. J. Jalview Version 2—a multiple sequence alignment editor and analysis workbench. *Bioinformatics* 25, 1189–1191 (2009).
2. Notredame, C., Higgins, D. G. & Heringa, J. T-Coffee: A novel method for fast and accurate multiple sequence alignment. *J. Mol. Biol.* 302, 205–217 (2000).
4. Jumper, J. *et al.* Highly accurate protein structure prediction with AlphaFold. *Nature* 596, 583–589 (2021).
5. Ide, D., Gorelik, A., Illes, K. & Nagar, B. Structural analysis of mammalian sialic acid esterase. *J. Mol. Biol.* 168801 (2024).
6. Dutschei, T. *et al.* Marine *Bacteroidetes* enzymatically digest xylans from terrestrial plants. *Environ. Microbiol.* 25, 1713–1727 (2023).
